# The Interstitial Duplication 15q11.2-q13 Syndrome Includes Autism, Mild Facial Anomalies and a Characteristic EEG Signature

**DOI:** 10.1002/aur.1284

**Published:** 2013-03-14

**Authors:** Nora Urraca, Julie Cleary, Victoria Brewer, Eniko K Pivnick, Kathryn McVicar, Ronald L Thibert, N Carolyn Schanen, Carmen Esmer, Dustin Lamport, Lawrence T Reiter

**Affiliations:** Department of Neurology, University of Tennessee Health Science CenterMemphis, Tennessee; Department of Pediatrics, University of Tennessee Health Science CenterMemphis, Tennessee; Department of Speech and Language Pathology, University of MemphisMemphis, Tennessee; Department of Neurology, Mass General HospitalBoston, Massachusetts; Human Genetics Research Laboratory, Nemours Biomedical ResearchWilmington, Delaware; Genetics Department, Hospital Central “Dr. Ignacio Morones Prieto,”San Luis Potosí, SLP, Mexico; Department of Psychology, University of South AlabamaMobile, Alabama; Department of Ophthalmology, University of Tennessee Health Science CenterMemphis, Tennessee

**Keywords:** autism, 15q duplication, imprinting, copy number variation, *UBE3A*

## Abstract

Chromosomal copy number variants (CNV) are the most common genetic lesion found in autism. Many autism-associated CNVs are duplications of chromosome 15q. Although most cases of interstitial (int) dup(15) that present clinically are *de novo* and maternally derived or inherited, both pathogenic and unaffected paternal duplications of 15q have been identified. We performed a phenotype/genotype analysis of individuals with interstitial 15q duplications to broaden our understanding of the 15q syndrome and investigate the contribution of 15q duplication to increased autism risk. All subjects were recruited solely on the basis of interstitial duplication 15q11.2-q13 status. Comparative array genome hybridization was used to determine the duplication size and boundaries while the methylation status of the maternally methylated *small nuclear ribonucleoprotein polypeptide N* gene was used to determine the parent of origin of the duplication. We determined the duplication size and parental origin for 14 int dup(15) subjects: 10 maternal and 4 paternal cases. The majority of int dup(15) cases recruited were maternal in origin, most likely due to our finding that maternal duplication was coincident with autism spectrum disorder. The size of the duplication did not correlate with the severity of the phenotype as established by Autism Diagnostic Observation Scale calibrated severity score. We identified phenotypes not comprehensively described before in this cohort including mild facial dysmorphism, sleep problems and an unusual electroencephalogram variant. Our results are consistent with the hypothesis that the maternally expressed ubiquitin protein ligase E3A gene is primarily responsible for the autism phenotype in int dup(15) since all maternal cases tested presented on the autism spectrum.

## Introduction

Autism spectrum disorders (ASDs) are a heterogeneous group of neurobehavioral syndromes characterized by deficits in social interaction, impairment in communication skills, and stereotypic and repetitive behaviors with onset before 3 years of age [American Psychiatric Association & American Psychiatric Association Task Force on DSM-IV, 1994]. Recent whole-genome analysis of *de novo* autistic cases suggests that as many as 1000 genes may be individually contributing to autism phenotypes [O'Roak, Deriziotis, et al., 2011; O'Roak, Vives, et al., 2012; Sanders et al., 2012; Vaags et al., 2012]. Both genetic and phenotypic heterogeneity have complicated the search for autism susceptibility genes, making the molecular analysis of autistic phenotypes challenging. However, recent diagnostic tests have made it possible to identify a genetic cause in ∼20–25% of cases [Miles, 2011]. The widespread use of array comparative genomic hybridization (aCGH) in the clinical setting has significantly accelerated the detection of submicroscopic microdeletions and microduplications, also known as copy number variants (CNVs), increasing the number of cases of autism associated with a detectable genetic lesion [Marshall & Scherer, 2012].

**Figure 1:**
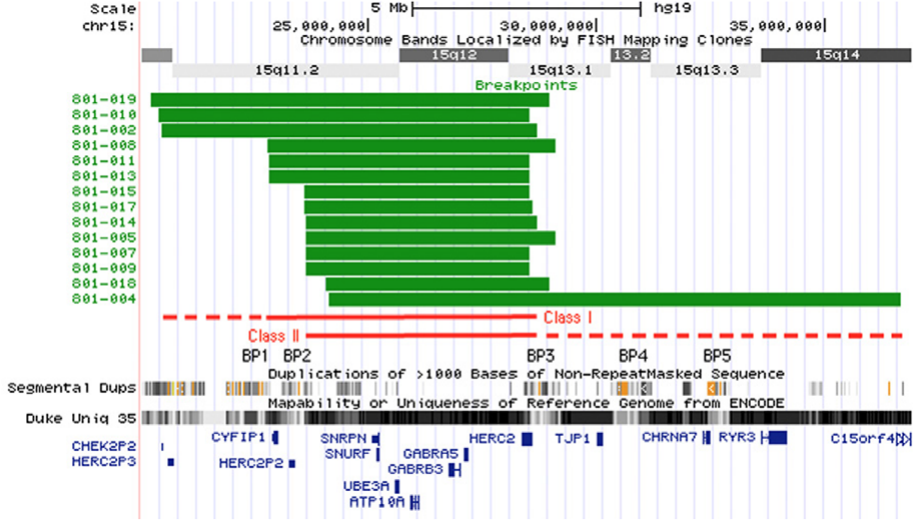
Size and breakpoints of interstitial duplication cases in this study. The boundaries of the duplications were determined using targeted or whole-genome array comparative genomic hybridization (aCGH) and the estimated base positions for the duplicated regions mapped to human genome assembly hg19 using the UCSC genome browser (green bars). Additional tracks included in this image are the ideogram track (showing cytogenetic bands), the segmental duplication track (showing the locations of segmental duplications >1000 bp) and the Duke Unique 35 track (showing the mapability of a given region of the genome). The locations of several significant RefSeq genes across the duplicated region are indicated in blue, but a comprehensive list of genes in this region is available at http://genome.ucsc.edu. The locations of canonical breakpoints for both interstitial and isodicentric duplications in this region are indicated as BP1–BP5. Note that these breakpoint locations coincide with the presence of an increased number of complex segmental duplications and a decrease in the mapability at this locus. Note the apparent gap in segmental duplications and mapability at the proximal end of the diagram. This is also the location of a gap in the hg19 alignment and is still unresolved. A more detailed view of both the proximal and distal breakpoints for these cases is available in Figure S1.

Several studies estimate that as many as 1–3% of all ASD cases may be the result of duplications of the 15q11.2-q13 region [Cook et al., 1997; Depienne et al., 2009; Veenstra-Vanderweele, Christian, & Cook, 2004; Vorstman et al., 2006]. A recent study using over 30 000 autism clinical cases to search for CNVs indicates that 15q duplications (combined interstitial and isodicentric 15q duplication) are the second most common duplication found in ASD with a frequency approaching 1 in 500 cases [Moreno-De-Luca et al., 2012]. Many of these CNV intervals are flanked by highly homologous low-copy-number repeats (LCRs), which can act as substrates for unequal meiotic exchange and recombination between nonallelic LCRs, resulting in microdeletion or microduplication [Devlin & Scherer, 2012; Stankiewicz & Lupski, 2010]. The 15q11.2-q13 region in particular is a site of frequent genomic rearrangements. In fact, *de novo* recurrent deletions of identical size occur in ∼70% of all Prader–Willi syndrome (PWS) and Angelman syndrome (AS) cases. When these deletions are paternally derived, the result is PWS, and when maternally derived, the individual will present with AS [Buiting, 2010]. The 4-Mb Prader–Willi/Angelman syndrome critical region (PWASCR) has a common distal breakpoint and two common proximal breakpoints that currently define the deletions as Class I and Class II (Fig. 1). Interstitial segmental duplications with these same breakpoints have also been described [Bolton et al., 2001; Roberts et al., 2002]. Class I deletions/duplications contain an additional set of nonimprinted genes including the *Fragile X associated cytoplasmic FMR1 interacting protein 1* (*CYFIP1*) gene and a copy of the *HECT* and *RLD* domain *containing E3 ubiquitin protein ligase 2 gene* (*HERC2*) at the boundary between Class I and Class II breakpoints.

Several groups have now reported individual patients with 15q11.2-q13 interstitial duplications (int dup (15)) that appear to show a parent origin effect with regards to the phenotype. Maternally transmitted 15q duplications show autistic features with variable degrees of developmental delay [Bolton et al., 2001], while only a few cases of paternally transmitted 15q duplication have been reported, and these cases primarily presented with no phenotype or speech delay and behavior problems [C. E. Browne et al., 1997; Cook et al., 1997; Mao et al., 2000; Mohandas et al., 1999; Veltman et al., 2005]. The prevailing hypothesis is that maternal duplications are associated with the ASD phenotype based mainly on individual case studies, while there is still an insufficient number of cases to establish the influence of paternal int dup(15) on the ASD phenotype [Hogart, Wu, LaSalle, & Schanen, 2010]. It is important to note, however, that no 15q duplications, paternal or maternal, show up in case controls in any of the current large autism cohorts, indicating that there is some pathogenicity to both maternal and paternal duplication [Moreno-De-Luca et al., 2012].

Our aim in this study was to perform an in-depth phenotype/genotype analysis of multiple individuals with interstitial 15q duplications to better describe the features of the syndrome in detail and to determine the relationship between int dup(15) and ASD diagnosis. We also identified new phenotypic characteristics of the interstitial duplication 15q syndrome involving facial appearance, neurological findings and sleep problems in this unique autism cohort.

## Methods

### Human Subjects

All subjects or their legal guardians provided informed consent, and all experiments on human samples were performed in compliance with the University of Tennessee Health Science Center Institutional Review Board. Subjects were recruited through the Dup15q Alliance (http://www.dup15q.org), a family support group for individuals with chromosome 15 duplications. Enrollment required a genetic testing report describing confirmed interstitial 15q duplication by targeted microarray or Fluorescent in situ Hybridization analysis. All int dup(15) subjects were recruited to the study with no prior knowledge of the parental origin of the duplication.

### Duplication Breakpoints and Parental Origin Status

Whole anticoagulated blood was collected for aCGH to determine the breakpoint boundaries of the duplication. The array platforms used were either a targeted SignatureChipOS array (*n* = 9, Signature Genomics, SPOKANE, WA, USA) or a Genome-Wide Affymetrix SNP 6.0 array (*n* = 5; Affymetrix, Santa Clara, CA, USA). Methylation-sensitive high-resolution melting (MS-HRM) curve analysis was used to determine the parent of origin of the duplicated chromosome as previously described [Urraca, Davis, Cook, Schanen, & Reiter, 2010]. MS-HRM is a post-polymerase chain reaction technique that can be used to profile methylation changes at the *small nuclear ribonucleoprotein polypeptide N* (*SNRPN*) promoter, which are parent of origin specific. An advantage of this method is that parental DNA is not required, so it can be run on all 15q duplication subjects, even if they were adopted.

### Subject Stratification, Autism and Neuropsychiatric Testing

As a general description of the cohort, our study subjects were categorized by gender, age ranges (preschool children 2–5.9 years, school-aged children 6–10.9 years and adolescents 11–17 years), parental origin (maternal or paternal) and breakpoint class using commonly accepted breakpoints for the PWS/AS deletion (I or II in Fig. 1). Based on the proximal breakpoint (BP1), if the duplication included this breakpoint it was called Class I, and if the duplication started at BP2, or distal to BP2, it was called Class II.

All evaluators were blinded to parent origin and class. A complete medical examination and a routine electroencephalogram (EEG) were performed for at least 45 min, with awake and sleep components when possible. Subjects were evaluated in person by a clinical geneticist. Two additional clinical geneticists evaluated facial dysmorphology using photographs of the subjects, with the majority (2/3) opinion being accepted as conclusive. Autism Diagnostic Interview-Revised (ADI-R) [Lord, Rutter, & Le Couteur, 1994] and Autism Diagnostic Observation Schedule (ADOS) [Lord et al., 1989] were applied by a research certified evaluator and used to classify the subjects as autistic, ASD or pervasive developmental disorder not otherwise specified. If the results of these instruments differed, clinical impression was used as the best determinant of autism status. Guidelines for selecting ADOS modules were followed and the appropriate module selected based on expressive language levels in each subject. An ADOS calibrated severity score (CSS) that allows for comparisons between different modules was also calculated, quantifying ASD severity across modules independent of age and verbal intelligence quotient (IQ) [Gotham, Pickles, & Lord, 2009]. A score of ≤3 is considered negative for autism, 4–5 is ASD and 6–10 is autism. Higher scores represent more severe autistic features. The Wechsler Preschool and Primary Scale of Intelligence—Fourth Edition or Wechsler Abbreviated Scale of Intelligence were attempted in all subjects depending on their age. Vineland Adaptive Behavior Scale-II (VABS-II) was used to evaluate everyday adaptive skills [Sparrow & Cicchetti, 1985]. The VABS-II has four domains: communication, daily living skills, socialization and motor skills, and an overall adaptive behavior composite standard score. Receptive vocabulary was assessed with the Peabody Picture Vocabulary Test, fourth edition (PPVT-IV) [Dunn, 2007]. Parents were also asked to complete two sleep questionnaires: Family Inventory of Sleep Habits (FISH) [Malow et al., 2009] and Children's Sleep Habits Questionnaire (CSHQ) [Owens, Spirito, & McGuinn, 2000]. The FISH is a scale developed to evaluate sleep hygiene in children with ASD [Malow et al., 2009]. The scale is from 12 to 60, with higher scores corresponding to better sleep habits. CSHQ is a parent report instrument with 33 items examining sleep behavior and symptoms of sleep disorders to identify sleep problems in children 4–12 years of age. It has a total score and eight subscales (bedtime resistance, sleep onset, sleep duration, sleep anxiety, night awakenings, parasomnias, sleep disorder breathing and daytime sleepiness). Higher scores reflect more sleep problems, and 41 points is the established cutoff to identify sleep problems in a subject [Owens et al., 2000].

### Statistical Analysis

The analyses were carried out with version 20.0 of the Statistical Package for the Social Sciences (IBM SPSS Statistics, Chicago, IL, USA) software. Statistical significance was set at a *P_value_* < 0.05. Differences among two groups were examined using the Mann–Whitney *U*-test. Correlation analyses were also done to test if any of the scales were dependent on age or IQ. We also looked if there was a correlation between ADOS severity score and sleep problems.

## Results

### Interstitial Duplication 15q Cohort

Fourteen subjects with a confirmed interstitial duplication encompassing the 15q11.2-q13 region were analyzed. Fluorescent in situ Hybridization or karyotype analysis was performed in all 14 subjects to rule out the presence of a marker chromosome 15. Eight males and six females were evaluated with a mean age of 88.4 ± 47.2 months with no age differences between groups with larger or smaller duplications, and the age range for the study was 3–16 years. There were six preschool children, four school-aged children and four adolescents. Several different ethnicities were represented in our cohort including subjects who identified themselves as Hispanic (1), Pacific Islander (1), African-American (2) and Caucasian (10). Most of the cases were *de novo* (11), with the exception of two siblings with an inherited paternal duplication (801-014 and 801-015) and one boy with an inherited maternal duplication (801-018).

### Duplication Boundaries and Parent of Origin

**Figure 2:**
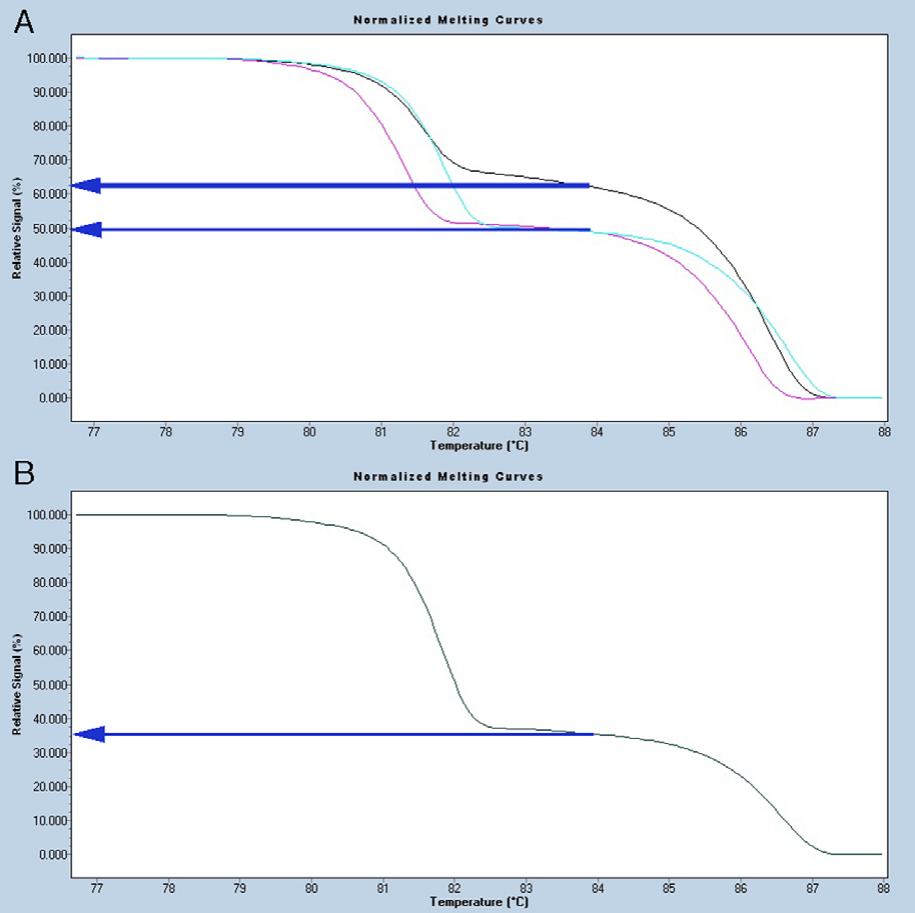
An example of a methylation-sensitive high-resolution melting (MS-HRM) result used to determine the parent of origin of the duplicated chromosome. (A) Family of a de novo maternally derived duplication (801-011), where the mother (pink) and the father (blue) have signal intensities in the normal range between 52–60%, and subject 801-011 (black) has a maternally derived duplication with signal intensity ≥66%. (B) A paternally derived duplication in subject 801-005, where parental DNA was unavailable. The signal intensity is ≤40% in this subject (black) and corresponds to a duplication on the paternal chromosome. More details on this method can be found in Urraca et al. [2010].

Six subjects in this study were designated Class I by aCGH, and eight have Class II interstitial duplications (Fig. 1). MS-HRM analysis indicated that ten subjects had maternal- and four had paternal-inherited or derived duplications (Table 1). An example of both a maternal and a paternal MS-HRM result are shown in Figure 2. All subjects in the study have duplications in the region between BP2 and BP3 that includes the PWS imprinting control locus (PWS-IC), a cluster of paternally expressed genes proximal to the PWS-IC, the maternally expressed *ubiquitin protein ligase E3A* (*UBE3A*) gene and a cluster of gamma-aminobutyric acid (GABA) receptor genes (Fig. 1). Three subjects (801-019, 801-010 and 801-002) had a larger than typical Class I duplication that extended into the centromeric region, resulting in the duplication of an additional eight Reference Sequence Database genes. One subject (801-004) had a novel 12.7-Mb duplication extending from BP2 well past BP5 and ending in the 15q14 region. The remaining subjects had canonical Class I (BP1–BP3) or Class II (BP2–BP3) duplications (Fig. 1). Duplication breakpoints in the region from the centromere to BP1 were extremely difficult to map because of an almost uninterrupted stretch of segmental duplications. In fact, there are very few unique sequences or single nucleotide polymorphisms to use for mapping these endpoints accurately at the proximal or distal ends of the duplication (Figure S1). For the purposes of phenotypic analysis, we considered the region between the centromere and BP1 as polymorphic and not contributing to the overall phenotype because expansions and duplications of these regions can occur in typically developing controls [Fantes et al., 2002]. Thus, duplications were classified in two genomic subgroups for phenotypic analysis, with six Class I duplications and eight in Class II including the subject with the larger duplication (Fig. 1).

**Table:** Interstitial 15q11.2-q13 Duplication Cohort Characteristics

Subject	Age	Sex	Autism	CSS	Full IQ	EEG	Class	Size (Mb)	Origin
801-002	7 years 11 months	M	ASD	6	79	Variant	I	8.2	Maternal
801-004	16 years 9 months	F	ASD	8	70	Normal	II	12.7	Maternal
801-005	6 years 3 months	M	No ASD	4	83	Variant	II	5.7	Paternal
801-007	12 years	M	Autism	9	Incomplete	Normal	II	5.0	Maternal
801-008	11 years	F	Autism	10	89	Normal	I	6.5	Maternal
801-009	3 years	F	Autism	10	Incomplete	Variant	II	5.0	Maternal
801-010	5 years 4 months	M	Autism	6	86	Variant	I	8.1	Maternal
801-011	6 years 8 months	F	ASD	3	71	Variant	I	5.8	Maternal
801-013	4 years 8 months	M	Autism	N/A	72	Normal	I	5.8	Paternal
801-014	11 years	M	Autism	9	100	Variant	II	5.0	Paternal
801-015	5 years 5 months	F	No ASD	3	77	Variant	II	5.0	Paternal
801-017	3 years 1 months	F	Not Tested	N/A	Incomplete	Variant	II	5.1	Maternal
801-018	4 years 9 months	M	Autism	9	Incomplete	Variant	II	5.3	Maternal
801-019	5 years 4 months	M	Autism	10	Incomplete	N/A	I	8.7	Maternal

ASD, autism spectrum disorder; CSS, calibrated severity score; EEG, electroencephalogram; IQ, intelligence quotient; N/A, not available.

### Neurological Examination

**Figure 3:**
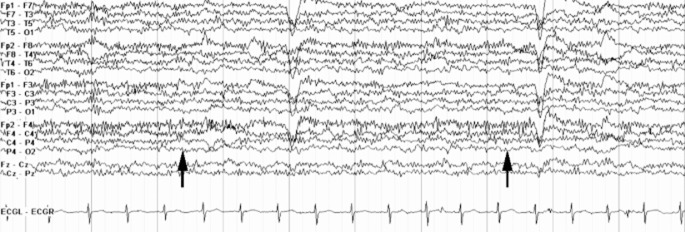
Excessive beta activity on electroencephalogram (EEG) in a child with an interstitial duplication of chromosome 15q11-q13. Arrows point to beta spikes in this example waking record EEG. This type of beta activity is typically seen in children on benzodiazapines, but none of the subjects were taking these medications.

Based on medical records and parent interview, nine out of 14 subjects had a history of hypotonia during infancy. Most subjects (*n* = 13) had developmental delay; five reached their infant language developmental milestones after their motor milestones. A third had a previous diagnosis of attention deficit hyperactivity disorder (ADHD) (Table 2). Of the 14 EEGs performed, 71% (10 subjects) had excessive diffuse 18–22 Hz beta spikes in the waking record (Fig. 3), while the remaining four had completely normal patterns. There was no correlation between the parent of origin and the beta variant, but three out of four subjects without the variant were older than 11 years of age. Subject 801-018 had epileptiform discharges on EEG, which presented as generalized spike and wave activity activated by photic stimulation.

**Table:** Clinical Details of Int Dup(15) Subjects

	Maternal origin										Paternal origin					Total	
Features	002	003	004	007	008	009	010	011	017	018	019	005	013	014	015	
Round face	–	+	–	–	+	N/A	–	+	+	–	N/A	–	+	+	+	7/13
Broad forehead	–	+	–	–	–	N/A	+	–	–	+	N/A	+	–	+	–	5/13
Long palpebral fissures	–	+	–	–	–	N/A	+	+	+	+	N/A	+	–	+	+	8/13
Nose, Short(S)/Long(L)	S	S	L	L	S	N/A	S	S	S	S	N/A	S	S	S	S	11/13
Wide nasal bridge	–	–	+	–	–	N/A	+	+	+	+	N/A	–	+	+	+	8/13
Bulbous nose	–	+	+	+	+	N/A	–	+	–	+	N/A	–	+	+	+	9/13
Anteverted nares	+	–	–	–	+	N/A	+	+	+	+	N/A	–	–	–	–	6/13
Short(S)/Long(L) philtrum	L	L	S	L	L	N/A	L	L	L	L	N/A	L	L	L	L	12/13
Thin upper lip	–	–	–	+	–	N/A	+	–	+	–	N/A	–	+	+	+	6/13
Pointed chin	–	+	–	–	–	N/A	+	–	–	+	N/A	+	–	–	–	4/13
Full cheeks	–	+	+	–	+	N/A	–	+	+	+	N/A	–	+	+	+	9/13
Wide Mouth	+	–	–	–	–	N/A	–	+	+	+	N/A	–	–	–	–	4/13
Other findings	RFM	OFA	–	H	DM/NLA	–	–	NLA	NLA/RFM	–	T	H/OFA	H/CHDAAG/NLA	–	–	
Neurological	H/AD	Ht	H/AD/OCD	AD	–	Ht	–	NT/A/Hp	Ht/Hp	H/BM/S	Ht	AD	–	Ht/AD/BM	Ht/AD	

H, hernia; T, torticollis; DM, diabetes mellitus; CHD; congenital heart defect; AG, abnormal genitalia; OFA, orbital facial asymmetry; NLA, nasal labial folds asymmetry; RFM, relax facial muscles; Ht, hypotonia; AD, attention deficit; OCD, obsessive-compulsive disorder; Hp, hemiparesis; BM, brain malformation; S, seizures; NT, neonatal tremors, A, apraxia; N/A, not available.

### Dysmorphology Evaluation

**Figure 4:**
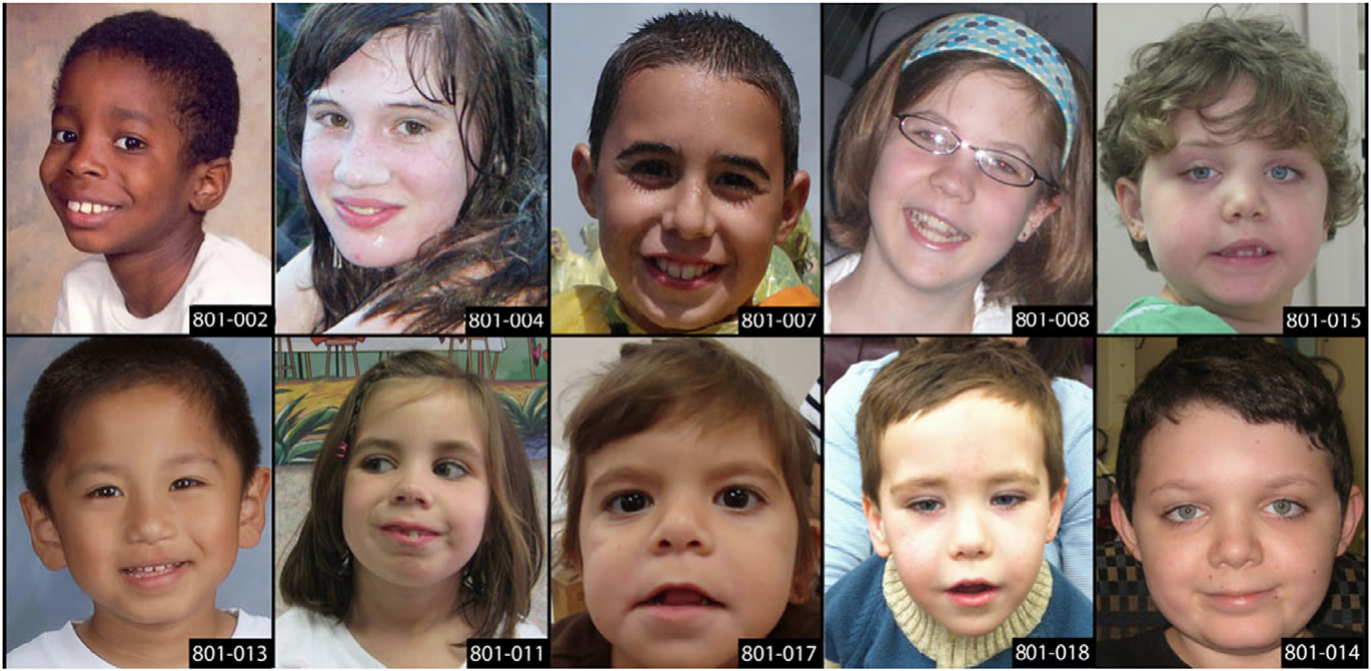
Facial features of int dup(15) subjects. Most individuals pictured are maternal duplication with the exception of subjects 801-013, 801-014 and 801-015. The two photos at the far right are from siblings 801-014 and 801-015. Note the long philtrum, shortened bulbous nose and full cheeks in most individuals. All subjects pictured consented to the use of their image for publication purposes in accordance with Institutional Review Board (IRB) policy.

We looked for subtle, but possibly characteristic facial features in 12/14 subjects with interstitial duplication of 15q. The dysmorphic features with an agreement between at least two of three clinical geneticists who viewed photos of the subjects are detailed in Table 2. The most frequent minor anomalies recognized were a long philtrum (94%), short nose (84%), bulbous nose (69%) and full cheeks (69%). There were no differences in the facial anomalies based on parent of origin (Fig. 4).

### Autistic Features

There were 11 subjects with an ASD/autism diagnosis, seven males and four females (1.75:1 ratio). Ten subjects (77%) were concordant between the ADOS and ADI-R. Among the remaining subjects who were discordant between ADOS and ADI-R, 801-005 and 801-015 were classified by clinical impression as nonautistic and 801-011 as autistic (Table 1). 801-005 was known to have ADHD causing ADOS scores to verge on ASD most probably because of metalinguistic difficulties related to working memory and open-ended oral questions being ignored because of inattention and oppositional behavior. 801-015 was smiling, had eye contact, presented a full affect and attended well during evaluation, in accordance with her nonautistic ADOS result. Although 801-011 was not autistic by ADOS, she clearly had stereotyped speech and was unable to initiate or maintain social interaction. It was not possible to complete the ADOS testing in 801-017 because this subject cried and was unable to be engaged long enough to complete the test. All 9/9 maternal and 2/4 paternal subjects scored as ASD or autism. The 11 autistic subjects showed no statistically significant differences for ADI-R and ADOS subdomains regardless of their breakpoints or size of the duplication. There was also no statistically significant difference in ADOS severity score among maternal Class I and Class II groups. ADOS/ADI-R domain scores, Calibrated Severity Score and modules used for each subject can be found in Table S1.

### IQ, Behavior and Language Testing

IQ scores were only available in nine subjects, while five were simply unable to complete the tasks required to obtain an IQ score. The full-scale IQ mean was 80.8 ± 9.8. Statistical analysis was not performed because of the small number of subjects in each group.

On average, all subjects were reported on the VABS-II adaptive behavior overall and domain scores as moderately low (−2 standard deviations (SD)) or low (−3 SD). VABS-II overall score positively correlated with performance IQ score (r = 0.695, *P* = 0.028). No significant differences were observed between maternal Class I and maternal Class II subjects (data not shown).

Maternal Class II subjects scored lower on the PPVT-IV for receptive language abilities (58.7 ± 28) than subjects with maternal Class I duplications (72.6 ± 29), but there was wide variability within the same duplication class making it difficult to establish differences.

The sleep hygiene habits in our subjects did not differ significantly between maternal and paternal groups (Table 3). However, both maternal and paternal subjects had a CSHQ mean over the cutoff for parents reporting sleep quality problems. Paternal subjects reported more sleep problems than maternal subjects, especially parasomnias (Table 3). However, because of the small sample size, it was not possible to establish statistically significant differences among subjects with different parent of origin duplications. There was no correlation between CSS and the sleep problems.

**Table:** FISH and CSHQ Scores for Int Dup(15) Subjects

Test	Group	Mean ± SD
FISH	Maternal	48.7 ± 6.2
	Paternal	54.5 ± 5.3
CSHQ total	Maternal	43.6 ± 7.4
	Paternal	56.8 ± 12.2
Bedtime resistance	Maternal	7.8 ± 2.7
	Paternal	9.0 ± 3.9
Sleep onset	Maternal	1.1 ± 0.3
	Paternal	2.5 ± 1.0
Sleep duration	Maternal	3.5 ± 1.0
	Paternal	6.0 ± 2.1
Sleep anxiety	Maternal	6.0 ± 2.1
	Paternal	7.0 ± 2
Night waking	Maternal	4.3 ± 1.3
	Paternal	6.8 ± 0.5
Parasomnias	Maternal	8.7 ± 0.9
	Paternal	14.5 ± 4.6
Sleep disorder (breathing)	Maternal	3.0 ± 0.6
	Paternal	4.3 ± 0.9
Daytime sleepiness	Maternal	11.7 ± 2.2
	Paternal	12.7 ± 1.9

FISH, Family Inventory of Sleep Habits; CSHQ, Children's Sleep Habits Questionnaire; SD, standard deviation.

## Discussion

Since the first report of a 15q duplication case including molecular data [Clayton-Smith, Webb, Cheng, Pembrey, & Malcolm, 1993], several individual case reports have been published, but a complete phenotypic description of the interstitial 15q duplication syndrome has never been established. We have assembled the largest single cohort of interstitial duplication 15q subjects (14 total) for a detailed phenotype/genotype analysis of the autism component of the syndrome and to define some common features of int dup(15) syndrome. A limitation in this study is the lack of sufficient power for meaningful statistical comparisons between maternal and paternal duplication subjects due to a small number of paternal int dup(15) subjects. We must point out, however, that both breakpoint groups and parent of origin groups could be useful in future studies for statistical analysis.

Here we have shown for the first time that maternally derived or inherited duplications of the region between BP2 and BP3 are sufficient to produce a phenotype on the autism spectrum in all nine maternal duplication subjects tested. We must also note that two out of four paternal duplication subjects examined also had autism (Table 1). Our particular cohort is too small to establish that paternal duplications do not cause autism. However, we do describe two paternal duplication siblings discordant for the autism phenotype (subjects 801-014 and 801-015), suggesting that the paternal duplication alone was not fully penetrant for an autism phenotype. In this family, only the boy met the criteria for autism classification (Table 1). Paternal int dup(15) individuals can present with autism [Bolton et al., 2004; Depienne et al., 2009]. Most striking in the current study was the finding that all maternal subjects were classified on the autism spectrum, indicating that a maternally expressed gene contributes to the autism phenotype. Autism has also been found more frequently in Prader–Willi subjects with maternal uniparental disomy than individuals with PWS from paternal deletion of 15q11.2-q13 [Cassidy, 1997], again implying that a maternal-specific gene or genes in this region increases autism risk. Finally, consistent with our findings in the current study, there are cases of inherited int dup(15) where the parent has no obvious autism phenotype due to inheritance of a paternal int dup(15), but the child who inherits this same duplication maternally has autism [Bolton et al., 2001; C.E. Browne et al., 1997; Cook et al., 1997].

We confirmed an autism diagnosis in 1.75:1 boys compared with girls in our int dup(15) syndrome cohort in contrast to the 5:1 ratio typically found in idiopathic autism cases [The Autism and Developmental Disabilities Monitoring (ADDM) Network, 2012]. Most likely, we did not observe this gender bias because the duplicated region is on an autosome. This lack of gender bias also distinguishes 15q duplication autism from idiopathic cases, emphasizing a role for the genes within the duplication in the etiology of the autism phenotype. There were no statistically significant differences in autism symptomatology as measured by subdomains of the ADOS between Class I and II duplications, as have been reported for the AS deletions [Peters, Horowitz, Barbieri-Welge, Taylor, & Hundley, 2012]. Thus, the inclusion or exclusion of the genes between BP1 and BP2, including the Fragile X associated *CYFIP1* gene, do not appear to influence the autism phenotype in int dup(15) cohorts.

These findings suggest that maternal-specific transcripts in the 15q11.2-q13 interval between BP2 and BP3 are responsible for the ASD phenotype. The strongest candidate gene is *UBE3A*, which is expressed only from the maternal allele in most neurons of the mammalian brain [Dindot, Antalffy, Bhattacharjee, & Beaudet, 2008; Herzing, Kim, Cook, & Ledbetter, 2001]. This gene has been proposed as a candidate gene for autism susceptibility in the past, although linkage and association studies have been inconsistent [Cook Jr et al., 1998; Guffanti et al., 2011; Nurmi et al., 2001]. Other than the *UBE3A* gene, the only other maternally expressed gene in the region is the *ATPase, class V, type 10A* (*ATP10A*) gene, which encodes an amphipathic phospholipid transporter and is most likely not involved in neurological phenotypes [Halleck et al., 1999; Herzing et al., 2001]. Studies in mouse models also indicate that *ATP10A* is not expressed exclusively from the maternal allele in neurons as previously proposed [DuBose, Johnstone, Smith, Hallett, & Resnick, 2010]. Furthermore, studies using human brain samples indicate that *ATP10A* expression is predominantly biallelic in brain and is influenced by both gender and genetic variation in the population [Hogart, Patzel, & LaSalle, 2008]. Here we propose that maternal interstitial duplications of the region between BP2 and BP3 are sufficient to produce an autism phenotype due most likely to overexpression of the maternally expressed *UBE3A* gene on the duplicated chromosomal segment.

We found that the size of the duplication did not correlate with the severity of the phenotype in several cases. Most notably, subject 801-004 had the largest (12.7 Mb) duplication that extended into the 15q13.3 region. This subject scored one SD below the average for the language test, and although the every day adaptive scores were low, none of the four domains were the lowest, as might be expected for such a large duplication which spans the 15q13.3 region. Individuals with 15q13.3 duplications have been reported with phenotypes from neurotypical behavior to developmental delay/intellectual disability and psychiatric disease [Szafranski et al., 2010]. Our subject had autism and mood liability when evaluated at 16 years of age. The 15q13.3 region includes the *cholinergic receptor, nicotinic, alpha 7 gene* (*CHRNA7*), which when deleted can be a risk factor for schizophrenia, according to association studies [Stephens et al., 2009]. Interestingly, our subjects with duplications extending proximally from BP1 performed with different grades of severity on PPVT, Vineland and CSS, and scored on average similar to those subjects with the BP1–BP3 duplications. These results indicate that additional dosage for the eight genes proximal to BP1 do not significantly affect the int dup(15) phenotype severity. In fact, some studies have indicated that centromeric expansions proximal to BP1 are common inherited polymorphisms and do not cause an appreciable phenotype [Fantes et al., 2002]. Our mapping data also suggest that the region between the centromere and BP1 contains such a high density of low copy repeats that the canonical breakpoint in this region (BP1) may be anywhere within the region (chr15:20 350 000–21 400 000) and buried in a sea of low copy repeats. Further high-resolution characterization of the reciprocal AS and PWS deletions in this region is warranted, and may help resolve the common recombination mechanism responsible for these genomic disorders.

Some phenotypic characteristics of isodicentric 15q (idic 15q) subjects, who are tetrasomic for the BP2–BP3 region, are shared among the int dup(15) individuals in our study. Individuals with int dup(15) have a more subtle phenotype, however, implying a gene dosage effect. The clinical features described in idic 15q syndrome are mild to profound developmental delay/intellectual disability, autism- or autistic-like behavior, central hypotonia, minor dysmorphisms and seizures [Battaglia, 2008]. A history of mild to moderate developmental delay was observed in 13 of our int dup(15) subjects. Hypotonia is found in almost all individuals with idic 15q duplications [Battaglia, 2005], while we observed hypotonia in more than half (60%) of our study population. In our study, only subject 801-018 had seizures (6%), which was most likely due to a periventricular leukomalacia that was not found in any other subjects and would not produce, on its own, the increased beta activity we described in other subjects (Table 2). Another subject could not travel to our study because of severe uncontrolled seizures, similar to the idic 15q phenotype [Battaglia, 2005]. Including these two individuals, the seizure frequency is 12.5% in the int dup(15) cohort as opposed to an estimated frequency of more than 75% in individuals with larger idic 15q duplications [Battaglia, 2008]. We suspect that the lower rate of seizures int dup(15) vs. idic 15q subjects is related to additional copies of a cluster of nonimprinted GABA receptor genes (including GABA A receptor, alpha 5) located within the common BP2–BP3 duplicated region (Fig. 1). Previous studies have established that these genes are not subject to imprinted expression in the brain and should therefore not be dependent on parental origin of the duplication to show elevated expression [Hogart, Nagarajan, Patzel, Yasui, & Lasalle, 2007]. In addition, studies done on brain samples from int dup (15) and idic (15) individuals did not show a significant correlation between GABAA expression and chromosome 15q copy number [Scoles, Urraca, Chadwick, Reiter, & Lasalle, 2011]. An EEG variant was observed in our cohort, but this pattern is of unclear clinical significance and is not indicative of a seizure phenotype. A similar pattern is typically seen in children treated with benzodiazepines or barbiturates, both of which promote GABA(A) receptors in the brain [T.R. Browne & Penry, 1973; Rudolph et al., 1999; Rudolph & Knoflach, 2011], but none of our subjects were on these medications. This EEG pattern appears to be specific to the presence of the BP2–BP3 duplication and is apparently not dependent on the parent of origin or ASD status of these individuals. Based on these observations, the most likely explanation for this anomaly is the presence of extra copies of the GABA receptor gene cluster. Expression studies of GABA levels in the brains of these subjects would be required to confirm our hypothesis. Interestingly, fewer adolescents had this EEG pattern thus raising the possibility that it may be an age-related finding as well. This characteristic EEG pattern has also been observed in idic (15) individuals being treated for seizure problems but is typically not seen in idiopathic autism cases where seizure is present (R. Thibert, personal observation). EEGs in this study were 1 hr in length. Ideally, a 24-hr EEG should be performed to gather additional data and ensure that a sleep component is captured. However, this was difficult to do logistically in the current study because most subjects traveled to the study site from out of state. Also, because this EEG pattern was an unexpected finding, we could not anticipate that a longer EEG would be beneficial.

We also observed that 15q duplication subjects have mild facial anomalies. There is variable expressivity among individual subjects as well as among family members [Bolton et al., 2001]. Previous studies described nondysmorphic subjects or minor anomalies, such as downslanting papebral fissures, full cheeks, and thin upper lip or full lips, but no feature has been consistent [Cohen et al., 2007; Orrico et al., 2009]. In our study, there was 46% agreement between the first two clinical geneticists, so a third geneticist described those discordant clinical features. One facial feature that showed 100% concordance among evaluators was the shape of the nose. The most frequent dysmorphic features were a long philtrum with a short bulbous nose, regions that develop embrionically from the frontonasal prominence. These mild facial dysmorphisms are not dependent on the parent of origin of the duplication and could be due to either *UBE3A* overexpression in nonimprinted tissues or some other gene/genes in BP2–BP3 region.

Half of all autistic children have at least one sleep problem [Krakowiak, Goodlin-Jones, Hertz-Picciotto, Croen, & Hansen, 2008]. Sleep onset problems, sleep duration problems, sleep anxiety and parasomnias are the most common issues [Couturier et al., 2005]. Although sleep problems are frequent in autism, they have been reported in only one previous 15q duplication study [Cook et al., 1997]. In contrast, on average, all of our subjects had sleep problems; although these issues were more severe in the few paternal subjects in the study due to the small sample size, this finding will require further analysis. Further sleep studies will be required to establish just how sleep is affected in duplication 15q syndrome and if it depends on the parental origin of the duplication.

In conclusion we observed that maternal duplications always produced an autism phenotype in a cohort recruited on the basis of duplication 15q11.2-q13 status alone. The ascertainment bias in the collection of paternal duplication subjects and the small number of subjects with paternal duplication that were analyzed makes it impossible to conclusively establish that paternal duplications of 15q do or do not increase autism risk. The greatest challenge is to determine if int dup(15) subjects present unique characteristics, allowing them to be distinguished clinically from the nonsyndromic autism. Unfortunately, the small number of int dup(15) cases available make it difficult to examine these variances from idiopathic autism. With the wide spread use of aCGH in the clinical setting, however, it may soon be possible to identify these int dup(15) individuals as a subgroup of autism. Future prospective studies will be necessary to provide more accurate genetic counseling to the family about the prognosis of the interstitial duplication 15q syndrome. The cohort described here is extremely valuable for the assessment of changes as these individuals develop through puberty and into adulthood. We anticipate that focusing on this cohort in the future will make it possible to establish new commonalities between int dup(15) and idiopathic autism.

## Supporting Information

Figure S1 Online resource. (A) Proximal and (B) distal breakpoints for int dup (15) subjects. University California Santa Cruz tracks being used are chromosome band, segmental duplications, mapability, HapMap single nucleotide polymorphisms (SNPs) and RefSeq Genes. Note the decrease in both mapability and the number of SNPs in regions covered by the segmental duplications. These duplications are located at the breakpoint boundaries for the duplications. In some cases, a single SNP may define the end of the duplication breakpoint, making the accurate mapping of duplication ends quite difficult and somewhat inaccurate within this region
