# Supplementary figures and images for: The Interstitial Duplication 15q11.2-q13 Syndrome Includes Autism, Mild Facial Anomalies and a Characteristic EEG Signature
Source: Autism Res. 2013 Mar 14;6(4):268–79. doi: 10.1002/aur.1284 (PMC3884762; doi:10.1002/aur.1284)

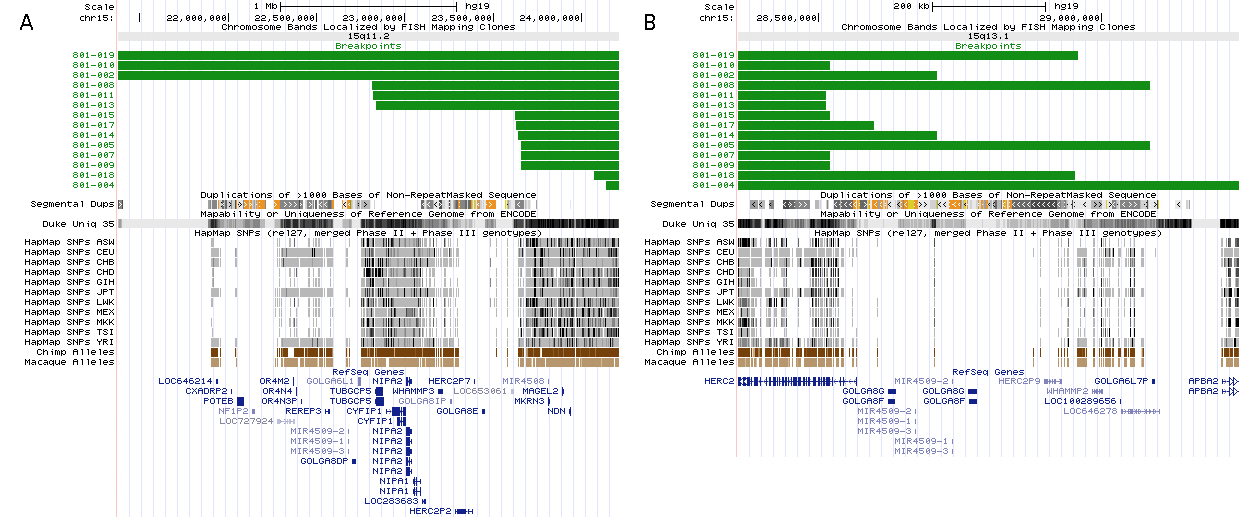

Supplement: Figure S1 — Online resource. (A) Proximal and (B) distal breakpoints for int dup (15) subjects. University California Santa Cruz tracks being used are chromosome band, segmental duplications, mapability, HapMap single nucleotide polymorphisms (SNPs) and RefSeq Genes. Note the decrease in both mapability and the number of SNPs in regions covered by the segmental duplications. These duplications are located at the breakpoint boundaries for the duplications. In some cases, a single SNP may define the end of the duplication breakpoint, making the accurate mapping of duplication ends quite difficult and somewhat inaccurate within this region [file aur0006-0268-sd1.tif]
